# Distal esophageal acid exposure and poor esophageal clearance correlate with probability of progression in Barrett’s esophagus as determined by the tissue systems pathology test

**DOI:** 10.1007/s00464-025-11930-y

**Published:** 2025-07-07

**Authors:** Sven E. Eriksson, Jennifer M. Kolb, Johnathan Nguyen, Inanc S. Sarici, Ping Zheng, Shahin Ayazi

**Affiliations:** 1 Foregut Division, Surgical Institute, Allegheny Health Network, Pittsburgh, PA USA; 2 Chevalier Jackson Research Center, Esophageal Institute, Western Pennsylvania Hospital, Allegheny Health Network, Pittsburgh, PA USA; 3 Vatche and Tamar Manoukian Division of Digestive Diseases, David Geffen School of Medicine at UCLA, Greater Los Angeles VA Healthcare System, Los Angeles, CA USA; 4 Department of Surgery, Drexel University, Philadelphia, PA USA; 5 Foregut Division, Surgical Institute, Allegheny Health Network, 4815 Liberty Avenue, Suite 454, Pittsburgh, PA 15224 USA

**Keywords:** Barrett’s esophagus, Esophageal pH-monitoring, High-resolution manometry, Progression, Dysplasia

## Abstract

**Background:**

The risk of progression from non-dysplastic Barrett’s esophagus (NDBE) to high grade dysplasia or esophageal adenocarcinoma (HGD/EAC) is low but variable. Biomarker assays can aid with risk stratification to optimize surveillance for NDBE. The role of diagnostic esophageal testing in prognosticating progression is unclear. The aim of this study was to evaluate whether esophageal physiology parameters correlate with a validated biomarker for BE risk progression.

**Methods:**

Patients with NDBE, including histology confirmed intestinal metaplasia < 1 cm, had their pathology specimen analyzed using a validated tissue systems pathology test with 9 biomarkers (TSP-9). This assay uses immunohistochemistry and digital pathology analysis to provide a 5-year risk of progression to HGD/EAC. These patients also underwent esophageal pH-monitoring and high-resolution impedance manometry (HRIM). Correlation analyses were performed between TSP-9 risk percent and esophageal testing.

**Results:**

A total of 59 patients [52.5% male; mean (SD) age 59 (14)] were included (40 NDBE, 19 < 1 cm IM) between 2021 and 2023. The median (IQR) TSP-9 value for 5-year risk of progression was low at 2.0% (2.0–3.0%). There were 8 (13.6%) statistical outliers with higher risk ranging from 5.0 to 10.0%. Risk of progression in the entire cohort was directly correlated with physiology testing parameters including DeMeester score (*R* = 0.30), acid exposure time (AET) (*R* = 0.34), duration of longest reflux episode on pH-monitoring (*R* = 0.30), and % incomplete bolus clearance on HRIM (*R* = 0.35) (*p* < 0.05 for all).

In a subgroup of 19 patients with < 1 cm IM, risk of progression had a stronger correlation with DeMeester score (*R* = 0.65), AET (*R* = 0.67), supine AET (*R* = 0.70), number of reflux episodes on pH-monitoring (*R* = 0.50) and % incomplete bolus clearance on HRIM (*R* = 0.68) (*p* < 0.05 for all).

**Conclusion:**

There was a direct correlation between 5-year risk of progression to HGD/EAC using TSP-9 and distal esophageal acid exposure and poor esophageal clearance among patients with NDBE that was even stronger in those with < 1 cm of IM. These findings suggest that esophageal physiology testing may have value in predicting risk progression in BE.

**Graphical abstract:**

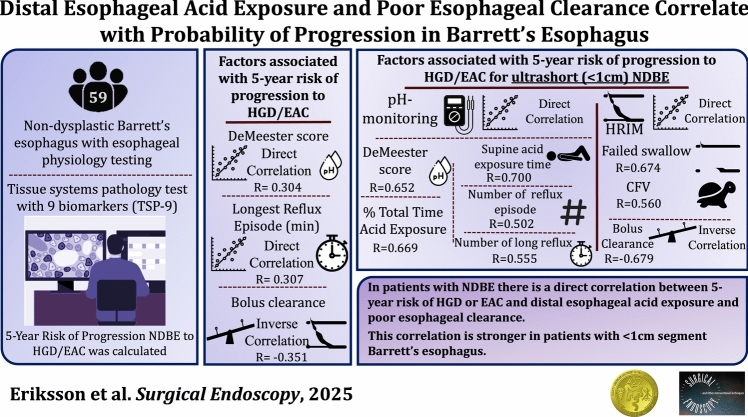

Barrett’s Esophagus (BE) is the precursor lesion to esophageal adenocarcinoma (EAC), and a known sequelae of chronic gastroesophageal reflux disease (GERD). Routine endoscopic surveillance with biopsies based on grade of dysplasia is recommended to identify patients early in the metaplasia-dysplasia-EAC sequence and intervene with endoscopic eradication therapy. However, disease progression from non-dysplastic BE (NDBE) to high grade dysplasia or EAC (HGD/EAC) is uncommon, and studies have suggested that surveillance may not be cost effective [1]. Furthermore, patients with < 1 cm of intestinal metaplasia (IM) are thought to have sufficiently low risk to potentially forego biopsy altogether. In the United States these patients do not meet the criteria or definition of BE and would not warrant surveillance [2]. In order to improve the effectiveness of early detection of EAC, studies have stressed the need to identify additional prognostic factors to further risk stratify this large group of patients with NDBE [1].

There is a paucity of data on the prognostic value of esophageal physiology testing in the assessment of risk of progression along the metaplasia-dysplasia-carcinoma sequence. Limited studies have suggested that patients with a higher DeMeester score and poor esophageal body peristalsis are more likely to progress from GERD to BE [3]. Whether or not these findings could be extrapolated to progression from NDBE to HGD/EAC or if the degree of distal esophageal acid exposure and esophageal body function could be useful in risk stratification is not known. The challenge with evaluating predictors and risk factors for BE progression is that development of cancer is rare, affecting an average of 3–4% of patients within 5 years of their initial diagnosis [4–7]. Therefore, case–control studies require a large number of patients with long-term follow up to identify factors that predict disease progression. Also, most studies continue to cite the same general, well described risk factors, such as BE segment length and degree of dysplasia, that are not very specific to the individual patient.

A novel, tissue systems pathology test with 9 biomarkers (TSP-9; Castle Biosciences, Pittsburgh, PA) can be used to circumvent these study demands and provide more precise prognostic information based on BE pinch biopsies. The TSP-9 assay uses immunofluorescent staining for biomarkers associated with disease progression, AI driven high-resolution digital pathology analysis and a validated prognostic model to determine an individual patient’s 5-year risk of disease progression to HGD/EAC [8]. The TSP-9 assay has been validated in several robust clinical studies and appears to have the highest predictive power for risk of disease progression [8–11]. Accordingly, TSP-9 can be used to help identify other factors associated with disease progression, and to test the hypothesis that esophageal physiology testing may play an important role in understanding the interplay between GERD, BE and risk of progression. Therefore, we designed this study to determine if pH-monitoring components and high-resolution impedance manometry characteristics correlate with the TSP-9 score for 5-year risk of disease progression from NDBE to HGD/EAC.

## Materials and methods

### Study population

This was a retrospective review of prospectively collected data from patients who underwent both TSP-9 assay testing and esophageal physiology testing at Allegheny Health Network (Pittsburgh, PA) hospitals between 2021 and 2023. The inclusion criteria were patients with NDBE or < 1 cm IM confirmed by expert pathologist, who were 18 years or older with no prior history of antireflux surgery and who completed esophageal physiology testing including 48-h pH-monitoring and high-resolution impedance manometry (HRIM). This study was evaluated and approved by the IRB of the Allegheny Health Network (IRB No. 2023-126).

### Clinical and objective evaluation

All patients underwent a comprehensive clinical evaluation with a focus on their esophageal and GERD symptoms. In addition, assessment included esophagogastroduodenoscopy (EGD) with biopsies and objective esophageal physiology testing performed on the same day, which included pH-monitoring and HRIM. On endoscopic evaluation, any amount of columnar lined esophagus was biopsied per the Seattle protocol [12]. Additionally endoscopic evaluation allowed for the assessment of esophageal dilation, tertiary contractions, esophagitis, liquid or food retention, and anatomical considerations such as the presence and size of a hiatal hernia. Biopsies were evaluated by an in-house expert gastrointestinal pathologist and only patients whose pathology results showed IM without dysplasia were sent out for the TSP-9 assay and included. Patients were categorized according to the length of histology confirmed IM as either meeting the definition of NDBE (≥ 1 cm) or < 1 cm IM.

### Tissue systems pathology with nine biomarkers (TSP-9) assay

**Fig. 1:**
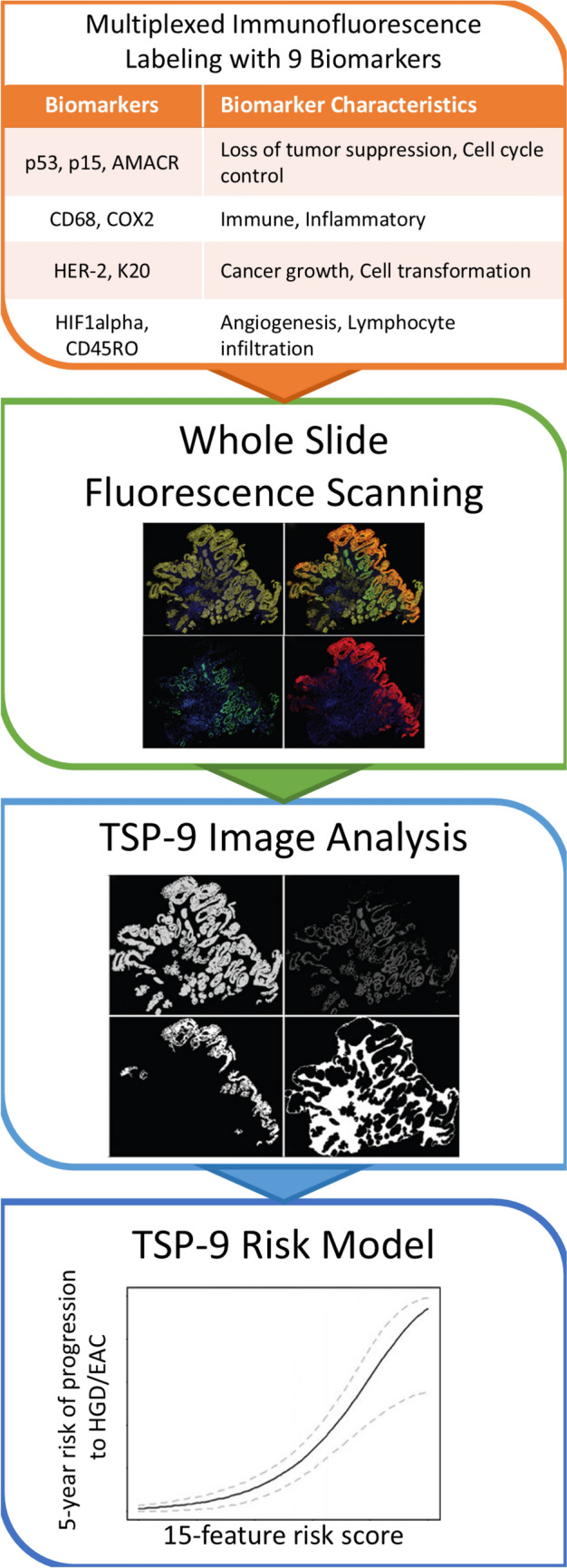
Diagram of the TSP-9 assay procedures. Specimens are first stained and labeled with 9 immunofluorescent biomarkers associated with an elevated risk of progression from Barrett’s esophagus to high grade dysplasia or esophageal adenocarcinoma (HGD/EAC). High-resolution fluorescent whole slide images are then digitally scanned and an artificial intelligence-driven image analysis algorithm is applied to detect cellular structures as well as frequency and intensity of biomarker immunofluorescence. These immunofluorescent and cellular structure data are processed into 15-features which comprise a weighted scaled risk score. Finally, a previously validated risk of progression model translates the risk score into the 5-year risk of progression to HGD/EAC

The TSP-9 assay is performed in several distinct phases: biopsy, immunofluorescent staining, artificial intelligence high-resolution digital pathological analysis, and prognostic modeling (Fig. 1). The assay can be run on routinely obtained biopsies and does not require any additional sampling. Specimens are sectioned and fixed with formalin, embedded with paraffin, stained with hematoxylin and eosin, and labeled using immunofluorescent stains for 9 biomarkers previously associated with risk of progression from BE to EAC. These biomarkers include loss of tumor suppression and cell cycle control markers (p53, p15 and AMACR), immune and inflammatory markers (CD68 and COX2), cancer growth and cell transformation markers (HER-2 and K20) and angiogenesis and memory lymphocyte infiltration markers (HIF1alpha and CD45RO).

High-resolution digital images at 20 × magnification are taken of the slides. Whole slide fluorescence images are analyzed using the TissueCypher™ Image Analysis Platform (Castle Biosciences, Pittsburgh, PA) as previously described [11]. Briefly, artificial intelligence-driven image data analysis algorithms are implemented with image mask techniques to detect tissue structures including stroma, epithelial nuclei, cells, epithelium, nuclei, cytoplasm and metaplastic epithelium. This cellular and subcellular data is used in conjunction with immunofluorescent data to identify features of morphology and intensity and frequency of biomarker expression.

The quantitative biomarker and morphology feature data are then used in a validated prognostic model based on multicenter matched case–control studies to determine the probability that the patient will progress to HGD/EAC within 5 years of their biopsy [8, 9]. This probability defined the TSP-9 risk of disease progression.

### pH-monitoring

During the EGD, a Bravo (Medtronic Inc, Minneapolis, MN) esophageal pH-monitoring capsule was placed 6 cm above the gastroesophageal junction. Patients were asked to stop taking any anti-secretory medications 10 days prior to and for the duration of testing. Distal esophageal acid exposure was recorded continuously for 48 h. Esophageal acid exposure was expressed by the standard parameters: acid exposure time (AET), AET in upright and supine positions, the number of reflux episodes, the number of reflux episodes lasting longer than 5 min, and the duration of the longest reflux episode. From these six values, a composite pH score (DeMeester score) was calculated. Abnormal distal esophageal acid exposure was defined as a DeMeester score > 14.7. The DeMeester score and its individual components were used to determine presence, severity and pattern of abnormal reflux.

### High-resolution impedance manometry

A trans-nasally placed 4.2 mm solid-state HRIM catheter with 36 pressure transducers spaced at 1 cm intervals and 19 impedance markers spread evenly in 2 cm increments between the copper coil sensors (Medtronic Inc, Minneapolis, MN). After calibration of the transducer, the procedures were performed in Fowler’s position with catheter positioned to ensure the entire esophagus was captured with three or more recording ports with intra-gastric location. Our standardized protocol consists of a baseline swallow-free recording of at least 3 consecutive respiratory cycles followed by ten consecutive liquid swallows. All HRIM files were analyzed using ManoView 3.3 analysis system (ManoView; Medtronic Inc., MN). High-resolution impedance manometry plots were interpreted in accordance with the Chicago Classification Version 4.0 [13]. The lower esophageal sphincter (LES) characteristics included: LES total length, LES abdominal length, LES resting pressure, and the integrated relaxation pressure (IRP). Esophageal body characteristics included: contractile amplitude, distal contractile integral (DCI), and percent bolus clearance. Additionally, the percent intact swallows (DCI 450–5000), weak swallow (DCI 100–450), and failed swallows (DCI < 100) were assessed as esophageal body characteristics. A percent bolus clearance ≤ 20% was considered poor bolus clearance.

### Statistical analyses

Demographic, clinical, individual pH-monitoring components and individual HRIM characteristics and the TSP-9 5-year probability of progression were collected and compared between patients with NDBE and < 1 cm IM. To determine the role of the antireflux barrier, LES competency and esophageal body function on disease progression in BE, the TSP-9 probability of progression was correlated with the DeMeester score and its individual components, the HRIM LES characteristics, and the HRIM esophageal body characteristics, respectively. Then these correlation analyses were repeated in the subgroup of patients with < 1 cm IM.

Data are expressed as median (interquartile range) or mean (standard deviation). Data points below the lower fence ($\text{first quartile }(Q1)-1.5*interquartile range$) or above the upper fence ($third quartile (Q3)+1.5*interquartile range$) were considered statistical outliers. Categorical variables were assessed using the Fisher exact test and continuous data using Wilcoxon signed rank test and Kruskal–Wallis tests as appropriate. The correlation analyses were performed using Spearman test and expressed as the correlation coefficient R with 95% confidence intervals (CI). A coefficient *R* ≥ 0.6 was considered strong and < 0.4 was considered weak. Statistical significance was defined as a *P* value < 0.05 for all analyses. All statistical analyses were performed using Statistical Analysis System (SAS) software (version 9.4, SAS Institute, Cary, NC).

## Results

**Fig. 2:**
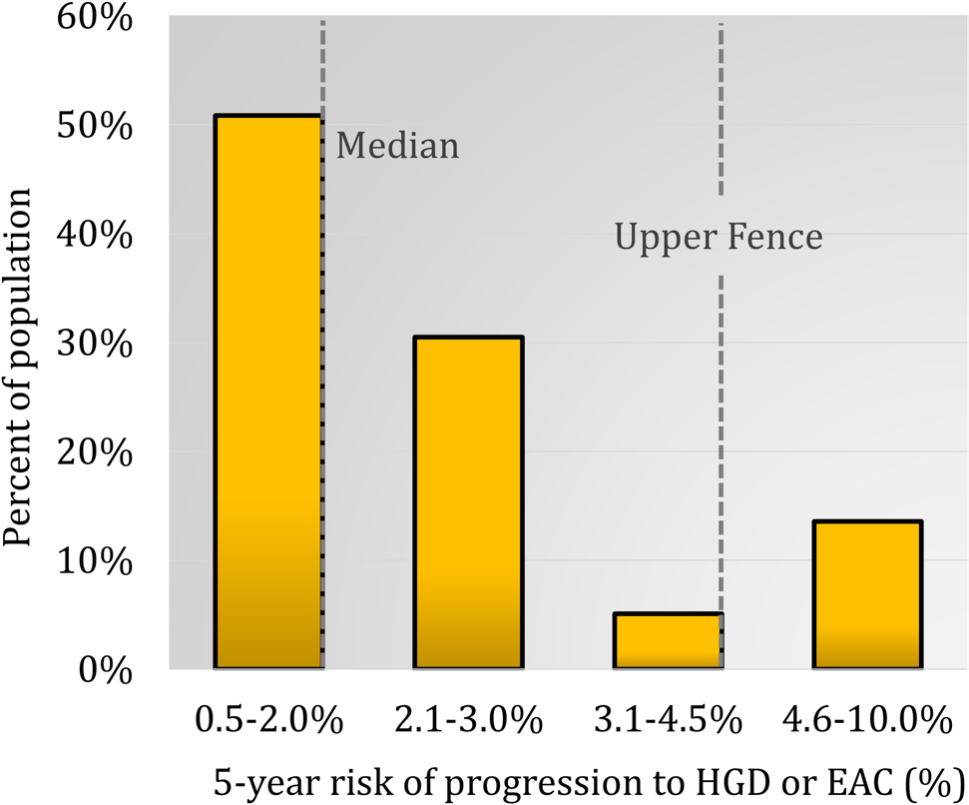
There was a bimodal distribution of the 5-year risk of progression NDBE or < 1 cm IM to HGD or EAC (%). The median (IQR) risk was 2.0% (2.0–3.0%), with a lower fence of 0.5% and an upper fence of 4.5%. There were no patients below the lower fence. The majority of patients were between the lower fence and the first quartile/median. Almost a third of the patients were between the median and the upper quartile. Only 5% of patients were between the upper quartile and upper fence. There were 14% of patients with risk above the upper fence. The maximum individual risk of progression in the population was 10%

The study population included 59 patients (mean age 59.4 (14), BMI 30.0 (7.3) and 50.9% male) with NDBE (68%) or < 1 cm IM (32%) who also underwent TSP-9 assay and esophageal physiology testing. Hiatal hernia was found in 84.8%. Los Angeles grade B, C or D erosive esophagitis was found in 22.0%. In the 40 patients with BE ≥ 1 cm, segment length measured a median (IQR) of 2.0 (1.0–3.5) cm with a range of 1 to 11 cm. The median (IQR) 5-year probability of progression to HGD/EAC based on TSP-9 was 2.00% (2.0–3.0%). There were 8 (13.6%) patients that were considered statistical outliers due to high-risk patients ranging from 5.0 to 10.0%. The histogram of 5-year risk of progression to HGD/EAC for the population is shown in Fig. 2.

### Disease progression and pH-monitoring

**Fig. 3:**
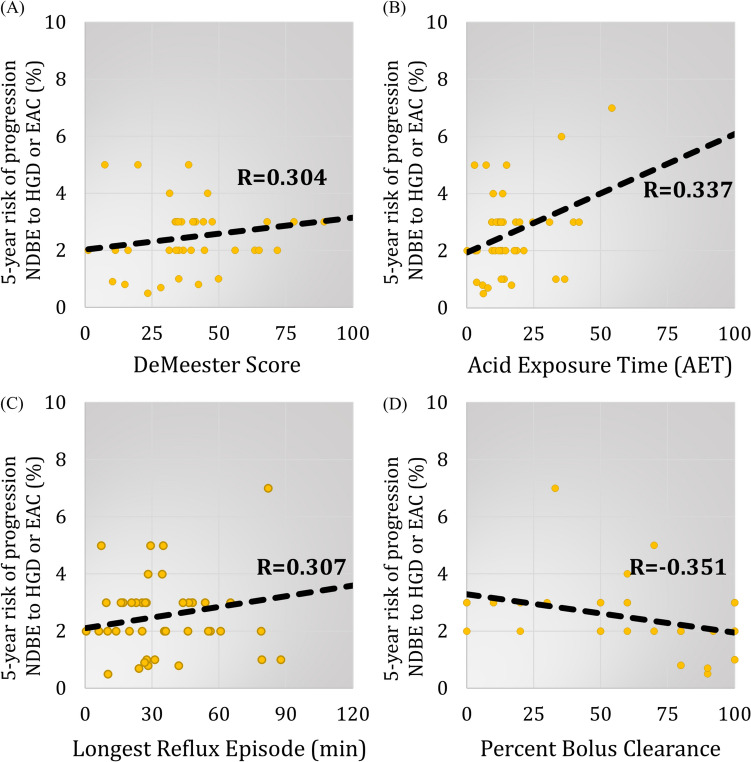
There were significant correlations between 5-year risk of disease progression from non-dysplastic Barrett’s esophagus (NDBE) or < 1 cm intestinal metaplasia (IM) to high grade dysplasia or esophageal adenocarcinoma (HGD/EAC) and **A** DeMeester score (*R* = 0.304, 95% CI 0.01–0.55, *p* = 0.045), **B** acid exposure time (AET) (*R* = 0.337, 95% CI 0.04–0.58, *p* = 0.025), **C** longest reflux episode (*R* = 0.307, 95% CI 0.01–0.55, *p* = 0.049) and **D** percent bolus clearance (*R* = − 0.351, 95% CI − 0.60– − 0.04, *p* = 0.028)

Higher 5-year risk of disease progression was seen with higher DeMeester scores (*R* = 0.304, 95% CI 0.01–0.55) (Fig. 3A). Risk of progression was also directly correlated with AET (*R* = 0.337, 95% CI 0.04–0.58) (Fig. 3B) and longest reflux episode (*R* = 0.298, 95% CI 0.01–0.55) (Fig. 3C) components. Upright and supine AET, number of reflux episodes, and number of long reflux episodes were not correlated with disease progression (*p* > 0.05).

### Disease progression and esophageal manometry

None of the LES characteristics were significantly correlated with disease progression (data not shown). Among esophageal body characteristics, there was an indirect relationship between risk of progression and percent bolus clearance (*R* = − 0.351, 95% CI 0.04–0.60, *p* = 0.028) (Fig. 3D). In addition, patients with poor (≤ 20%) bolus clearance had a significantly higher risk of disease progression [3.0% (2–3) vs 2.0% (1–3), = 0.038]. None of the other esophageal body characteristics were correlated with disease progression (*p* > 0.05).

### Subgroup analysis for < 1 cm IM

There were 19 (32.2%) patients with < 1 cm IM. These patients were similar in age, sex, BMI, and presence of hiatal hernia or esophagitis (*p* > 0.05) to the other 40 patients. The risk of disease progression in this subgroup ranged from 0.5 to 7.0% and was significantly lower compared to patients with ≥ 1 cm NDBE [median 2.0% (0.9–3.0) vs 3.0% (2.0–3.0), *p* = 0.040]. Among patients with < 1 cm IM, 10.5% had ≥ 5% risk of disease progression.

**Fig. 4:**
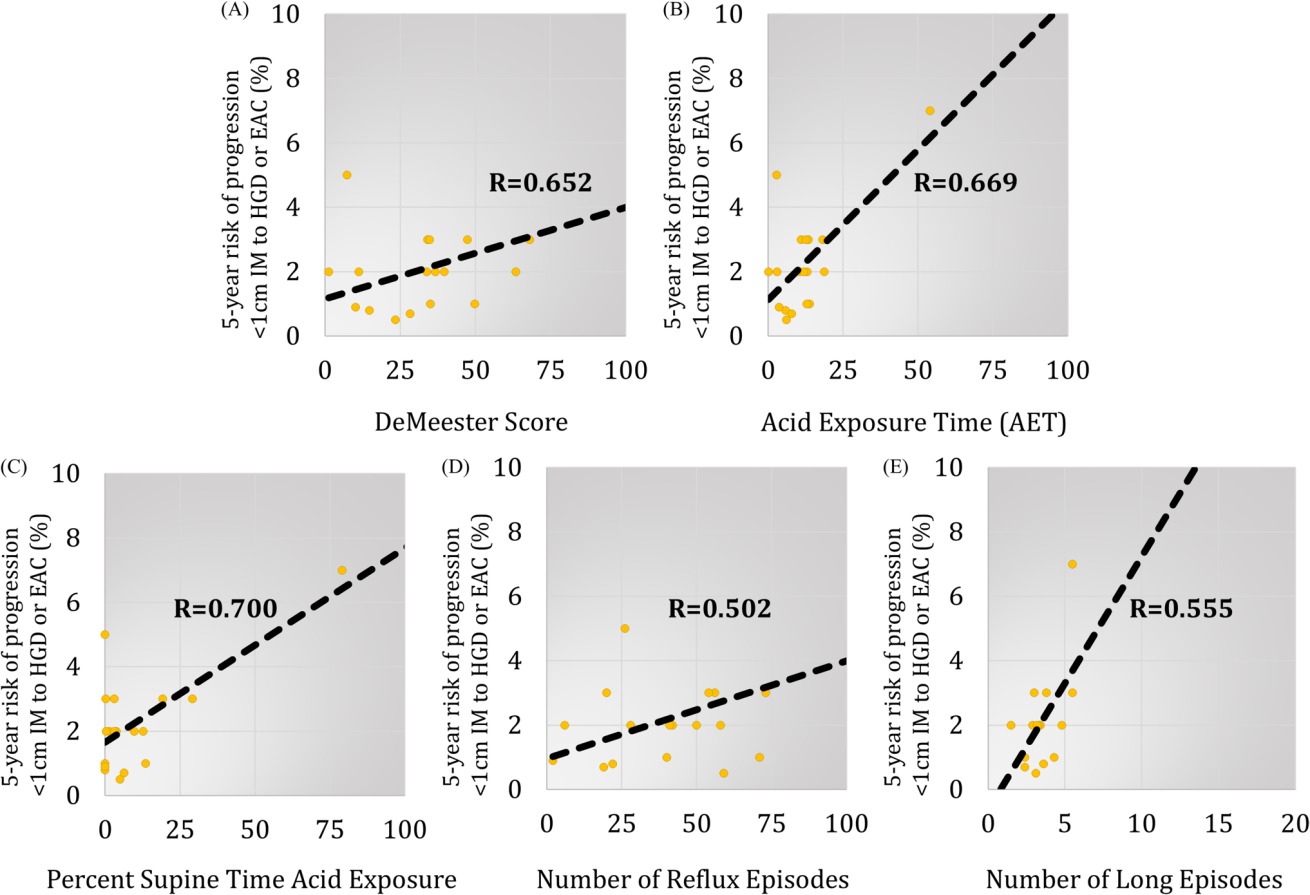
There were significant correlations between 5-year risk of disease progression from < 1 cm intestinal metaplasia (IM) to high grade dysplasia or esophageal adenocarcinoma (HGD/EAC) and **A** DeMeester Score (*R* = 0.652, 95% CI 0.27–0.86, *p* = 0.003), **B** acid exposure time (AET) (*R* = 0.669, 95% CI 0.29–0.86, *p* = 0.002), **C** supine AET (*R* = 0.700, 95% CI 0.35–0.88, *p* = 0.001), **D** number of reflux episodes (*R* = 0.502, 95% CI 0.05–0.79, p = 0.032) and **E** number of long reflux episodes (*R* = 0.555, 95% CI 0.12–0.81, *p* = 0.015)

Results from pH-monitoring showed fewer abnormalities in the < 1 cm IM group compared to the NDBE group with lower DeMeester score, fewer reflux episodes, and less AET (Table 1). The correlation analysis showed a strong and significant association between the DeMeester score and the 5-year risk of progression to HGD/EAC in the < 1 cm IM group (*R* = 0.65, 95% CI 0.27–0.86, *p* = 0.003). Among individual pH-monitoring components, there was also a strong significant direct correlation with AET (*R* = 0.67, 95% CI 0.29–0.86, *p* = 0.002), supine AET (*R* = 0.70, 95% CI 0.35–0.88, *p* = 0.001). Number of reflux episodes (*R* = 0.50, 95% CI 0.05–0.79, *p* = 0.032) and number of long reflux episodes (*R* = 0.56, 95% CI 0.12–0.81, *p* = 0.015) were also directly correlated (Fig. 4).

**Table 1:** Comparison of pH-monitoring components between < 1 cm IM and NBDE groups

Characteristic	< 1 cm IM (*n* = 19)	NDBE (*n* = 40)	*p* Value
AET, mean (SD)	12.2 (11.7)	18.6 (10.9)	0.03
Upright AET, mean (SD)	12.4 (7.1)	18.7 (8.8)	0.04
Supine AET, mean (SD)	10.4 (18.9)	16.9 (17.7)	0.07
Number of reflux episodes, mean (SD)	43.3 (27.0)	76.2 (45.0)	0.01
Number of long (> 5min) reflux episodes, mean (SD)	7.2 (5.8)	10.8 (5.8)	0.06
Longest reflux episode, mean (SD), mins	33.2 (23.3)	46.3 (39.4)	0.25
DeMeester Score, mean (SD)	39.4 (37.4)	60.5 (37.3)	0.02
Abnormal DeMeester Score (> 14.7), %	77.8%	100.0%	0.02

*IM* intestinal metaplasia, *NDBE* non-dysplastic Barrett’s esophagus, *AET* acid exposure time, *SD* standard deviation

**Fig. 5:**
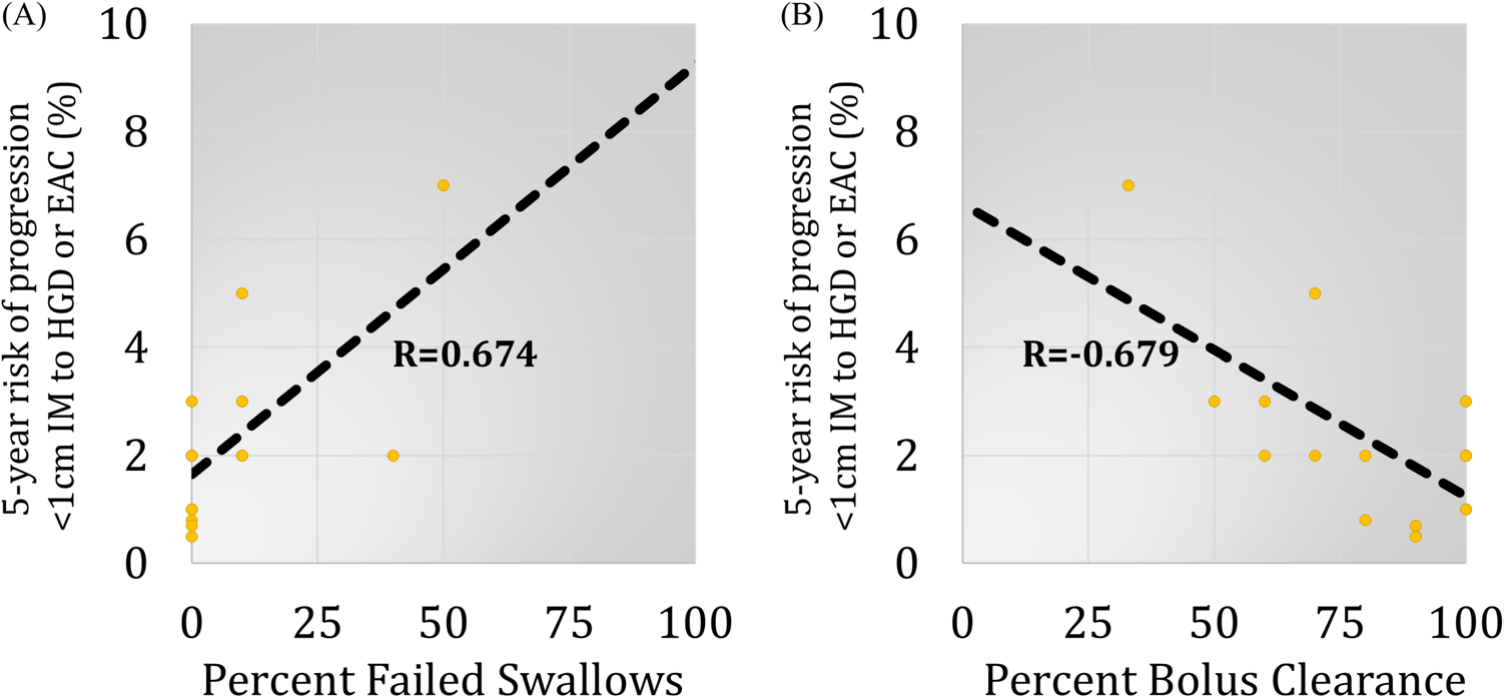
There were significant correlations between 5-year risk of disease progression from < 1 cm intestinal metaplasia (IM) to high grade dysplasia or esophageal adenocarcinoma (HGD/EAC) and **A** percent failed swallows (*R* = 0.674, 95% CI 0.27–0.88, *p* = 0.003) and **B** percent bolus clearance (*R* = − 0.679, 95% CI − 0.82 to − 0.06, *p* = 0.003)

The individual manometric characteristics are compared between < 1 cm IM and the rest of the population in Table 2. Patients with < 1 cm IM had significantly better bolus clearance (80.2% vs 44.0%, *p* = 0.003). All other manometric characteristics were comparable between groups. The LES characteristics did not correlate with TSP-9 probability of progression. Among esophageal body characteristics, TSP-9 probability of progression was directly correlated with percent failed swallows (*R* = 0.67, 95% CI 0.27–0.88, *p* = 0.003) (Fig. 5A) and indirectly correlated with percent bolus clearance (*R* = − 0.68, 95% CI − 0.82 to − 0.06, *p* = 0.003) (Fig. 5B). None of the other esophageal body characteristics were correlated with disease progression (*p* > 0.05).

**Table 2:** Comparison of high-resolution impedance manometry characteristics between < 1 cm IM and NBDE groups

Characteristic	< 1 cm IM (*n* = 19)	NDBE (*n* = 40)	*p* Value
Lower esophageal sphincter (LES) characteristics			
LES resting pressure, mean (SD), mmHg	22.8 (13.0)	21.3 (17.2)	0.41
LES total length, mean (SD), cm	3.1 (1.3)	2.6 (1.0)	0.35
LES abdominal length, mean (SD), cm	1.2 (1.3)	0.9 (1.0)	0.48
Integrated relaxation pressure, mean (SD), mmHg	9.5 (6.7)	7.9 (6.4)	0.38
Esophageal body characteristics			
Distal contractile integral, mean (SD), mmHg cm s	2.0k (1.8k)	1.3k (1.0k)	0.21
Contractile amplitude, mean (SD), mmHg	83.8 (37.9)	65.6 (30.0)	0.11
% Intact swallows, mean (SD)	77.0 (31.2)	62.3 (35.3)	0.14
% Failed swallows, mean (SD)	8.8 (15.0)	17.7 (26.5)	0.34
% Weak swallows, mean (SD)	9.9 (17.5)	18.7 (29.7)	0.91
% Bolus clearance, mean (SD)	80.2 (21.1)	44.0 (37.0)	< 0.01

*IM* intestinal metaplasia, *NDBE* non-dysplastic Barrett’s esophagus, *SD* standard deviation

## Discussion

Early detection of HGD or cancer in patients with known BE offers the best chance to change the course of the disease and improve outcomes. This paradigm requires high quality endoscopic screening and ongoing surveillance to optimize dysplasia detection in high-risk patients, which has to be balanced against labor and cost intensive over-testing in low-risk patients. An efficient and effective surveillance program would stratify patients according to the likelihood of their disease progressing and tailor surveillance intervals and follow-up care to their individual risk. The availability of novel prognostic biomarkers may facilitate a de-escalation of surveillance in a lower risk population. In the present study, we used the validated TSP-9 assay in 59 patients with NDBE, of whom 19 had < 1 cm IM, and found a variable 5-year probability of progression to HGD/EAC ranging from 0.5 to 10.0%. These results are consistent with previously published studies showing that the majority of NDBE patients are at low risk for disease progression. Notably, this study demonstrated the association of esophageal physiology findings with the TSP-9 results. Specifically, risk of disease progression in BE was directly correlated with degree of acid exposure and indirectly correlated with esophageal clearance. Esophageal manometry and pH-monitoring are routinely performed during the workup of patients with chronic GERD and often overlap with evaluation of BE. Therefore, assessment of esophageal physiology factors through these tests may be an effective and practical means to help risk stratify BE patients. These findings complement recent efforts to develop non-invasive risk stratification strategies for BE, including cytologic samples and molecular biomarkers [14].

The 5-year probability of disease progression reported by the TSP-9 assay provides prognostic data at the time of biopsy. It is based on data from patients who were followed longitudinally after their endoscopy and several subsequent validation studies. The initial TSP-9 assay was modeled using multi-institutional case–control data obtained from 79 BE patients with disease progression and 287 BE patients with no progression after a median of 5.6 years [8]. Patients were randomized into model training and validation groups. The resultant model was highly predictive of progression (HR 9.42, *p* < 0.0001), was validated (C-index = 0.772) and even outperformed predictions based on pathological diagnosis, segment length, and p53 overexpression [8]. A similarly designed subsequent study again found that TSP-9 was highly predictive of disease progression (OR: 46.0, *p* < 0.0001) and outperformed expert GI pathologists [9]. Additionally, an age, sex, BE segment length and pathologic diagnosis matched case–control study found that TSP-9 was a predictor of disease progression (*p* < 0.0001) [10]. Subsequent large cohort multicenter studies have corroborated these findings. A study of 552 patients (472 with NDBE), of whom 152 had disease progression at a median of 6.5 years, found that high risk on TSP-9 was an independent predictor of disease progression to HGD/EAC (OR: 6.0, *p* < 0.001) [15]. These studies demonstrate the validity of the TSP-9 assay as a prognostic tool that accurately translates to disease progression rates and can be used for risk stratification in patients with NDBE [16].

We found a strong relationship between increased distal esophageal acid exposure and probability of BE progression, suggesting that both the severity and duration of reflux may contribute to advancement along the GERD-NDBE-dysplasia-cancer continuum. This is consistent with prior studies demonstrating association between acid burden and histologic progression. A comparative analysis of 633 patients with reflux disease found a stepwise increase in AET and number of reflux episodes when moving from NERD to erosive esophagitis, to BE, and ultimately to HGD/EAC (*p* < 0.0001 for both) [17]. There is also indirect evidence that reducing acid exposure through pharmacotherapy may serve to reduce the risk of BE progression. In a study of 236 Veterans with NDBE followed for 1170 patient-years, PPI use was associated with a 75% reduction in the risk of dysplasia (HR: 0.25, *p* < 0.0001) [18]. Similarly, a meta-analysis comprising 155,769 patients found that PPI use reduced the odds of progression from BE to HGD/EAC (OR: 0.47, 95% CI 0.32–0.71) [19]. Surgical intervention may confer similar benefit: in a cohort of 140 patients with NDBE, those who did not undergo antireflux surgery were 2.3 times more likely to progress to dysplasia [20]. These studies suggest a direct relationship between degree of acid exposure and disease progression, consistent with our findings. The proposed mechanism underlying this relationship is that acid acts as a carcinogen in patients with BE. A study of 6 patients with BE who underwent biopsy before and after esophageal perfusion with HCl found that acid exposure results in formation of intracellular reactive oxygen species and double strand breaks in DNA, supporting a biologically coherent mechanism for carcinogenesis [21].

Among individual pH-monitoring components, supine AET and number of long episodes components were most strongly associated with risk of progression in our cohort, particularly in patients with < 1 cm of intestinal metaplasia. When elevated together these components suggest difficulty clearing refluxate without the aid of gravity, leading to prolonged mucosal-acid contact. Prior studies have described similar patterns. Studies have found associations between BE and higher DeMeester score (*p* = 0.03), AET (*p* < 0.002) and supine AET (*p* < 0.002) compared to GERD controls [22]. Other studies have demonstrated that patients with BE have higher DeMeester score, AET and more long reflux episodes [3, 23, 24]. This finding suggests that degree of acid exposure is elevated in BE independent of severity of esophagitis, consistent with our finding that risk of disease progression was correlated with degree of acid exposure, but not severity of esophagitis. These studies not only support the conclusion that risk of disease progression is correlated with the degree of acid exposure, but also show that there is a particularly strong relationship in patients with supine reflux and many long reflux episodes, consistent with our findings. These findings suggest that risk of disease progression may be more likely provoked by prolonged acid exposure than by frequent short reflux episodes. Further research into the relationship between different patterns of reflux and risk of disease progression is warranted.

Failure to clear a reflux episode in patients with poor bolus clearance results in a prolonged exposure of their non-dysplastic IM to carcinogenic acidic gastric refluxate. Consistent with this concept, we found that poor bolus clearance was correlated with risk of disease progression. This explanation is consistent with a study comparing distal esophageal acid clearance capacity between patients with BE, esophagitis and healthy controls. They found that patients with BE had significantly lower acid clearance capacity (*p* < 0.001) [23]. Acid clearance capacity has been demonstrated to be a product of saliva production and esophageal body function, specifically bolus clearance. However, saliva production is unaffected by BE. Therefore, this study suggests that there is a relationship between worse bolus clearance and BE, consistent with our findings. Studies looking at manometry and BE have found similar associations. A multicenter cohort study of 84 patients with NDBE who underwent manometry and subsequent endoscopy at 2.1 years found that the 12 patients with disease progression only had 52% bolus clearance, significantly worse than the 100% bolus clearance in the non-progression group [25]. Collectively these studies suggest that risk of progression from NDBE to HGD/EAC is related to both degree of acid exposure and adequacy of the esophageal body to clear reflux when it occurs.

In addition to poor bolus clearance, risk of disease progression was also correlated with percent failed swallows, a marker of poor esophageal body contractility. Previous studies have also found an indirect relationship between esophageal body contractility and BE disease progression. A study of 84 patients with NDBE who underwent subsequent endoscopy at 2.1 years found that poor esophageal body contractility was an independent predictor of progression to dysplasia (OR1.2, *p* = 0.04) [25]. Another study of 58 patients with GERD (21 with BE) who underwent esophageal manometry found that patients who developed BE had worse esophageal body function, with lower contractile amplitude (*p* = 0.003) and percent peristalsis (81% vs 94%, *p* = 0.037) [3]. Additionally, a study comparing BE patients to patients with moderate and severe esophagitis, found that patients with BE had significantly lower contractile amplitudes (36 vs 41 vs 45 mmHg) [24]. These studies all suggest that as esophageal body function worsens, so too does the risk of BE and disease progression.

As expected, the overall 5-year risk of progression from NDBE to HGD/EAC was low, with an average risk of only 2.74%. Additionally, the majority of patients did not vary far from the median, with an interquartile range of just 1.0%. However, 13.6% of patients had a substantially higher risk than the majority, ranging from 5 to 10%, which should not be ignored. Meta-analysis of 2694 patients with low grade dysplasia found the pooled annual rate of progression to HGD/EAC to be 1.73%, which is equivalent to a 5-year risk of 8.36% [26]. Our results indicate that within a NDBE population, a substantial minority of patients have similar risk of progression as patients with LGD. Previous studies have also found this heterogeneity of risk among patients with NDBE. A study of 699 (56.1% NDBE) patients with BE, of which 190 progressed to HGD/EAC, found that rate of disease progression in NDBE patients with a high TSP-9 risk percent was similar to the rate of progression in expert pathologist diagnosed LGD (3.2%/year vs 3.7%/year) [10]. A matched case–control study similarly found that patients with NDBE and a high TSP-9 risk percent were more likely to progress to HGD/EAC than patients with expert pathologist diagnosed LGD [10]. These findings are clinically significant as multiple society guidelines recommend surveillance at 3–5 years for NDBE, but for LGD endoscopic eradication therapy or a shorter surveillance interval ranging from 3 to 12 months are recommended [27–30]. Therefore, it is necessary to further risk stratify patients with NDBE in order to optimize management and outcomes. The TSP-9 assay provides a risk assessment specifically to aid in this type of risk stratification.

The present study suggests that esophageal physiology data may be used to fill this risk assessment need as well. We found the strongest correlation between physiology data and risk of disease progression in patients with < 1 cm IM. Patients with < 1 cm IM also had significantly lower risk than patients with ≥ 1 cm NDBE. However, even within the < 1 cm IM group, 11% of patients had substantially higher risk of disease progression than expected given their relatively favorable pathology. This is a particularly important finding as recent United States professional society guidelines have recommended against taking biopsies of columnar lined esophagus < 1 cm, but recommend surveillance every 3–5 years for NDBE [30]. Given the overall lower risk in patients with < 1 cm IM, this finding does not imply that all patients with < 1 cm IM should be biopsied. However, it does suggest that a small subgroup of high-risk patients may benefit from a lower threshold for biopsy. The challenge is identifying these patients with higher risk. The strong correlation between physiology and risk of disease progression in this population suggests that the presence of severe reflux and poor bolus clearance may be used to identify the patients who may benefit from a lower threshold for biopsy. These findings suggest that while routine physiology testing is not indicated solely for risk stratification, results of pH-monitoring and manometry, which are often already available in patients undergoing foregut evaluation, may provide additional prognostic insight. In patients with BE, particularly those with < 1 cm IM, identifying severe acid exposure or poor bolus clearance could inform individualized surveillance strategies and highlight those who may benefit from closer follow-up.

Progression from BE to HGD/EAC is difficult to study, due to its low incidence. As a result, most studies are limited to a small number of disease progression cases. Obtaining comprehensive esophageal physiology testing in this population compounds the challenge of acquiring an adequate sample size. This study used validated prognostic modeling to quantify risk of progression in a large patient cohort. This unique approach enabled the acquisition of adequate data to be the first study to correlate esophageal physiology testing parameters with individual patient disease progression risk. In addition to these strengths, we also acknowledge several limitations of this study, including limited sample size and the single center design. At our center, we recommend physiology testing for all patients with chronic reflux symptoms as part of a comprehensive foregut evaluation. However, the presence of endoscopic evidence of severe reflux, such as LA grade C/D esophagitis or extensive BE may be sufficient evidence of GERD to forego subsequent pH-monitoring. This practice may be the reason that despite the association between degree of acid exposure and risk of disease progression, esophagitis was not associated with worse risk. This was also a cross-sectional study using prognostic risk at the time of physiology testing. Prognostic risk is not absolute. However, our prognostic risk of progression from NDBE to HGD/EAC of 2.74% is consistent with a meta-analysis of 6,847 patients with NDBE whose disease progression rate was equivalent to a 5-year rate of 2.95% [5]. This suggests that the relationship between esophageal physiology and disease progression is reliable. Nevertheless, large cohort long-term prospective studies looking at baseline esophageal physiology components and characteristics as predictors of actual disease progression are warranted in patients with NDBE as well as with low grade dysplasia,

## Conclusion

Barrett’s surveillance represents a balancing act between maximizing the detection of pathology in patients at high risk of disease progression and minimizing the number of low-risk patients who undergo repetitive negative examinations. Given the devastating consequences of detecting esophageal cancer late and the high costs of endoscopy, there is a need for additional prognostic factors that correlate with disease progression to guide a more personalized management strategy. In this study, we used the validated TSP-9 assay in a cohort of 59 patients with NDBE (19 of whom had < 1 cm IM) to determine their 5-year risk of progression to HGD/EAC to be 2.0%. However, a substantial minority of patients had risks ranging from 5.0 to 10.0%, highlighting the heterogeneity of risk despite the favorable pathologic diagnosis. Risk of disease progression was directly correlated with degree of acid exposure and poor esophageal bolus clearance. These results suggest that pH-monitoring and esophageal manometry data may prove useful to aid risk stratification to better identify patients who may need an escalated surveillance schedule. Further prospective studies are needed to validate the use of esophageal physiology testing in long-term risk prediction, but the current evidence supports its potential role in optimizing patient care and improving outcomes in Barrett’s esophagus.
